# Carnegie Honor for a Physician

**Published:** 1908-04

**Authors:** 


### Carnegie Honor for a Physician

SO infrequently is the medical profession materially recognised that the recent announcement of the granting by the Carnegie Foundation for the Advancement of Teaching of a pension of $3,000.00 to Dr. Stanford B. Chaillé, of Tulane University, New Orleans, attracts attention and arouses universal commendation.

Dr. Chaillé is one of the veteran workers in medical education in the United States, having on the 20th of March completed his fiftieth year as a teacher; and it is a fitting recognition of his faithful work as an educator, that he was awarded the highest retiring pension at the command of the fund. There have been only a few of these maximum pensions allowed since the establishment of the fund, and this is the first instance where a physician has been the recipient. Not alone is Dr. Chaillé to be congratulated on the public recognition of his work in so material a manner, but the medical profession is distinctly honored.

THE wide-awake and hustling, if somewhat careless, drug merchant in the city of Washington, D. C., who made much money and considerable local fame by his exploitation of such popular remedies as "Cuforhedake" and "Brane-Fude," has the distinguished honor of being the subject of the first recorded legal conviction under the Food and Drugs law.

This gentleman, Robert N. Harper, by name, is not alone a druggist of many years' experience, but he is interested in many of the commercial enterprises of Washington and occupies a rather more exalted position than the ordinary small retail drug merchant, who has neither special backing nor influence. Mr. Harper is president of a bank and has other financial interests quite as prominent. He is also something of a social light, and counted among his friends some of the best known men in Washington.

None of these attainments, however, saved him from the conviction which followed a careful trial of the case which, on its face and in spite of the obstacles thrown up by the defendant's attorneys, was rather clear cut.. There is a provision for a fine in the penalty in connection with imprisonment, and the importance of the case and the attention it has attracted may be judged by the fact that it is generally reported in the daily newspapers, that the President has directed the district attorney to work for imprisonment as the penalty, and to not quietly submit to the imposition of the mere maximum fine of $500.00 which would not materially affect Mr. Harper's bank account, and certainly not his mental attitude toward the law.

---

The Buffalo Department of Health under the direction of Dr. Ernest Wende is again publishing a monthly review of the sanitary conditions existing in Buffalo, and the first number of the new series was published January 31, 1908. Although curtailed considerably from the old monthly Bulletin, yet it contains a mass of interesting information which any Buffalo physician and sanitarian should be acquainted with. It is a pity that sufficient appropriation was not made for publishing the old Bulletin, because it contained reports from all the heads of the department and publicity seems to be the order of the day. Those who were wont to criticise every official for neglect of duty, could see by the old Bulletin just what the officers of the health department were doing, and conversely perhaps these same officials were cognisant of the fact that their acts were being scrutinised—all of which tends to better the service under observation. The new Bulletin is edited by Dr. Franklin C. Gram, Registrar of the department and copies may be secured for the asking.

---

An American physician, writing from Berlin, (*N. Y. Tribune*) says: "I had the good fortune to be present at the 'Aerzetekommers,' arranged in honor of Dr. Robert Koch. It took place in the great hall of the royal opera house last night—February 11—and was such a doctors' meeting as I never saw before and the like of which I shall never see in our country. There were two thousand physicians present. At the table of honor with Koch sat the men who had worked with him, Proskauer, Rubner, Gaffiky, Frosch and Wassermann, and there were professors, 'geheimrats,' 'geheime medezinalräte' 'obermedezinalrate' and at least one 'ministerial direktor.' Next to Koch sat Geheimrat Körte, who took part with the others in the Kaiser salamander, with which the evening began, although he had celebrated his ninetieth birthday a few days before. There were some serious addresses, full of tributes to the scientist, who had but recently returned from the far South crowned with new success in his field. A memorial medal was presented also, but the men came there to have a good time, and they had it. I heard physicians who stand high in the profession sing, recite humerous poems, play on instruments and even take part in grotesque play. It was an invigorating spectacle. The hit of the evening was a one-act farce by Dr. Alfred Peyser, the scene of which is laid in the court of Dr. Koch's present home in Berlin. The time is December 11, 1993, the 150th birthday of Koch. The peep into the future gave the authors of a number of songs a fine opportunity to make queer predictions. One of the best songs was entitled 'The Wail of the Bacillus.' "

---

Dr. Frank Van Fleet, chairman of the committee on legislation of the Medical Society of the State of New York, informs the medical profession that the perennial optometry bill is up again and will be voted on in a few days. Among other bad provisions of the bill it authorises a Board of Examiners whose only qualifications shall be a knowledge of optics.

Every member of the state society should write his senator and assemblyman at once insisting that this bill be killed. If not familiar with the names of senators and assemblymen write the clerk of the senate and the speaker of the assembly.

